# Synaptic bouton sizes are tuned to best fit their physiological performances

**DOI:** 10.1186/1471-2202-12-S1-P371

**Published:** 2011-07-18

**Authors:** Markus Knodel, Gillian Queisser, Dan Bucher, Romina Geiger, Lee How Ge, Alfio Grillo, Christoph Schuster, Gabriel Wittum

**Affiliations:** 1 GCSC Frankfurt, Frankfurt University, Germany; 2 IZN Heidelberg, Heidelberg University, Germany

## 

**Figure 1:**
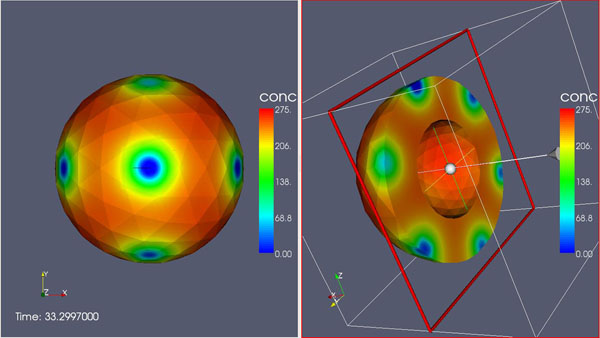
Stimulated Bouton, Simulation

To truly appreciate the myriad of events which relate synaptic function and vesicle dynamics, simulations should be done in a spatially realistic environment. This holds true in particular in order to explain the rather astonishing motor patterns presented here which we observed within in vivo recordings which underlie peristaltic contractions at a well characterized synapse, the neuromuscular junction (NMJ) of the Drosophila larva. To this end, we have employed a reductionist approach and generated three dimensional models of single presynaptic boutons at the Drosophila larval NMJ. Vesicle dynamics are described by diffusion-like partial differential equations which are solved numerically on unstructured grids using the uG platform. In our model we varied parameters such as bouton-size, vesicle output probability (Po), stimulation frequency and number of synapses, to observe how altering these parameters effected bouton function. Hence we demonstrate that the morphologic and physiologic specialization maybe a convergent evolutionary adaptation to regulate the trade off between sustained, low output, and short term, high output, synaptic signals. There seems to be a biologically meaningful explanation for the co-existence of the two different bouton types as previously observed at the NMJ (characterized especially by the relation between size and P_o_),the assigning of two different tasks with respect to short- and long-time behaviour could allow for an optimized interplay of different synapse types. As a side product, we demonstrate how advanced methods from numerical mathematics could help in future to resolve also other difficult experimental neurobiological issues. Figure 1.
